# Thyroid Extract for Myxœdema

**Published:** 1892-07-16

**Authors:** 


### The Practitioner's Mirror and Retrospect.

[*The Editor will be glad to receive offers of co-operation and contributions from members of the profession. All letters should be addressed to* The Editor, The Lodge, Porchester Square, London, W.]

### MEDICINE.

### THYROID EXTRACT FOR MYXŒDEMA.

It may be that cases of myxœdema are actually more numerous than heretofore, but it is quite certain that many cases were formerly unrecognized as a definite organic disease, or at any rate as a clinical entity. Recently, attention having been directed to the disease, its relative frequency has been abundantly demonstrated.

That the exhibition of pilocarpin has some beneficial effect in the disease is well known, and a course of massage combined with the use of this drug has, up to the present, constituted a recognised method of treating this affection, highly unsatisfactory as it is.

Before reviewing the few cases in human patients in whom the thyroid gland of lower animals has been made to serve as a therapeutic agent, we may briefly state what is at present known as to the functions of the thyroid glands, and for this we turn to Mr. Victor Horsley's recently-published remarks on the subject.* It is unnecessary to quote his summary of the different views, mostly pure hypotheses, which have been, or are, held as regards the function of the thyroid. Referring to the views that its function is directly or indirectly hæmopoietic, Mr. Horsley writes: "There is no evidence that the thyroid gland is directly hæmopoietic as regards the red corpuscles or the blood plasma, but Kohlrausch has found that in the acini are bodies closely resembling blood plates. This introduces us at once to the eighth hypothesis, according to which the thyroid gland is indirectly hæmopoietic. It has always seemed to me obvious that its influence must be exercised first on the chemical metabolism, especially of the albuminoid constituents of the blood, and that while there was no positive evidence that the normal effect is a constructive one, it is perfectly clear that the absence of the gland must very markedly alter the constitution of the blood, and so, indirectly, hæmopoiesis must be dependent on the integrity of the thyroid. This is a point of considerable importance in view of the fact that all observers who have examined the condition of the blood after thyroidectomy, have found it notably altered in its constitution and function, even apart from the changes in the corpuscles. The changes in the blood, apart from the alteration in the numbers of the corpuscles, are as follows: (*a*) Increased venosity; (*b*) Great diminution in the amount of oxygen, which may in the arterial blood fall below the normal proportion in the veins. This condition has been termed by Herzen "anoxyæmia"; (*c*) Presence of abnormal constituents of the plasma, *e.g.*, mucin. The ninth hypothesis, which directly connects the function of the gland with the functions of the female sexual organs, is many centuries old. Of late years the subject has been so carefully considered by Freunel, in its chief bearings, that it is hardly necessary for me here to do more than point out how the truth of the conjunction of the functional activities of these parts is confirmed by the far greater liability of the female sex to suffer from myxœdema. The harmony between the two sets of organs is evinced by the enlargement of the thyroid occurring when active changes happen in the sexual organs. . . There remains yet the tenth hypothesis, which is the most important of all, and the demonstration of which is necessary to the proof that the thyroid gland is the active agency in metabolism"; *i.e.*, that the gland modifies or destroys substances which, circulating in the blood, are harmful to the general economy, and then that it secretes some substance useful to the general metabolism of the body.

That fatal thyroidectomy in human beings and in some of the lower animals is practically certain to be followed by cachexia strumipriva, or myxœdema, may be taken as proved.

Mr. Horsley reminds us that the thyroid gland is in functional activity before birth, and is of special metabolic importance in early extra-uterine life, while its value falls as the general vital processes decrease. "Cachexia strumipriva has been found by all observers to occur with far greater frequency when the thyroidectomy has been performed in young individuals."

An examination of a table of 39 cases given by Bourneville shows that the liability to constitutional disturbance following the loss of the function of the thyroid gland is extremely marked in early life, and almost abruptly ceases at about the thirtieth year.

We may now turn to the therapeutical employment of the thyroid gland in the treatment of myxœdema. In a recent article we referred to Mr. Horsley's suggestion that the progress of myxœdema might be arrested by transplantation (into the peritoneal cavity) of a portion of the thyroid gland of a sheep, and reminded our readers that Professor Horsley's proposal has been put in practice by M. Lannelongue, who reported the case at the Paris Biological Society.

We have not seen any further report of this case, M. Lannelongue believed at the time that the operation had succeeded, but M. Chauveau thought it probable that the engrafted thyroid would be absorbed.

Drs. Harris and Wright have been successful in treating a case of myxœdema with thyroid gland grafting. The grafts were taken from a monkey. Their patient, which was a typical case of myxœdema, was kept in hospital, and chiefly in bed, for a month before operation. On April 4th a curved incision was made below each breast, and the glands were raised a little from the pectoral muscles. Half the thyroid gland from a small young green monkey, which was chloriformed to death in the ante-room, was then placed under each breast. The wounds were closed and healed by primary union. On the 29th she was sent to a convalescent hospital much better. The improvement was maintained, and she was sent home for some weeks. She relapsed very much into her former condition and was re-admitted. The authors remark that they fear the temporary improvement must be put down entirely to better hygienic conditions, and so far as their case goes, that no conclusion in favour of the procedure could be claimed. But Dr. Macpherson* has been successful in treating a case by transplantation of portions of the thyroid of a sheep. He found almost instantaneous relief from some of the most pronounced symptoms after the grafting had been performed, and he believes that a considerable portion of the gland which he grafted was ultimately vascularised in the body of his patient. For this reason he believes his method to be better than that of injecting liquid. His patient was 39 years of age, and three years before had manifested symptoms of myxœdema, which had become very pronounced. She was placed in bed for a week previous to the operation, and kept under the most favourable conditions. The right lobe of the sheep's thyroid was removed with the utmost antiseptic precautions possible, and after removal, washed in a warm, weak solution of perchloride of mercury. The lobe of the theroid was divided transversely into two equal parts, and each half was

* *Brit. Med. Journ.*, Jan. 30, 1892; *ibid*, Feb. 6, 1892.

* *Edinburgh Medical Journal*, May, 1892; *Therapeutic Gazette*, June 1892.

then split longitudinally. Two half-curved incisions were made in the infra-mammary region of the patient, through the skin and subcutaneous fat. A portion of the excessive fat having been removed, a piece of the gland, one and a half inches long, was placed in each wound in such a way that the surface of the split gland were in contact with the raw surfaces of the flaps of the wounds.

The results of the operation were as follows: (1) within twelve hours there was such a marked mental improvement in the patient as to be noticeable by every person who came in contact with her. She became talkative, cheerful, answered questions readily, and the mental reflex, which was formerly so slow, became altered and quickened. Her intelligence and spontaneity markedly increased. Her speech, however, remained slow and drawling. (2) The average daily temperature for seven days after the operation was 99·2 deg., and for nineteen days afterwards 98·9 deg. (3) The average daily qnantity of urine passed for nineteen days subsequent to the operation was forty-one ounces. (4) The fear at night, the melancholia and delusions disappeared on the day following the operation, and have not since recurred. (5) The vertical headache, more or less persistent for three years, has not since been felt. (6) The anæmic condition has been removed. (7) The skin is now soft and smooth, and the hair not so dry. (8) The quantity of urine passed for the twenty-four hours ending on February 28th was fifty-two ounces. (9) Menstruation, which had for three years been irregular, is now of monthly occurrence, and lasts only three days. In conclusion, Dr. Macpherson does not claim that his operation has removed the myxœdematous condition, but it has relieved the symptoms, which, with the exception of the melancholia and stupor, have all been more or less constant for three years. Under favourable conditions, he believes there is no reason why the relief should not continue. These favourable conditions are: (1) Warmth, upon which Dr. Byrom Bramwell insists in his very able and lucid article on "Myxœdema" in his "Atlas of Clinicsl Medicine." (2) A farinaceous dietary, according to Mr. Victor Horsley's recent researches (referred to in the early part of our article). (3) Avoidance of worry and overwork, which most authorities are agreed in recognising as a causative agency. (4) Perhaps massage over the gland, which Professor Grainger Stewart and Dr. John Thomson have used with success in one case, might with advantage be practised.

It must be remembered that in man and to a still more marked extent in dogs and other animals there are accessory glands which are capable by themselves in some cases in carrying on the ill-understood functions of the thryoid gland.

*(To be continued.)*

